# The influence of ambient cure chemistry and stoichiometry on epoxy coating surfaces†

**DOI:** 10.1039/d2ra05067f

**Published:** 2022-10-10

**Authors:** Callum Bannister, Alan Guy, Ralitsa Mihaylova, Joseph Orgill, Stephanie L. Burg, Andrew Parnell, Richard L. Thompson

**Affiliations:** Department of Chemistry, Durham University Durham UK; Safinah Ltd Gateshead UK; Department of Physics and Astronomy, University of Sheffield UK

## Abstract

The surface properties of epoxy resin coatings influence their function as substrates for subsequent coats. Variation in ambient cure conditions (temperature and relative humidity, RH), stoichiometry (ratio of epoxy: amine) and delay time between epoxy component mixing and film casting (“induction time”) significantly altered the surface properties of ambient cured epoxy resin coatings (Dow Epoxy Novolac D.E.N. 431, resorcinol diglycidyl ether and 4,4-diaminodicyclohexylmethane). Gravimetric analysis showed that increasing induction time significantly reduced surface layer formation (carbamation) of cured epoxy resin coatings at 80% RH but had no measurable effect at 40% RH and below. RMS surface roughness increased with increasing RH and decreased with increasing induction time and ambient cure temperature, at two stoichiometric extremes. However, the net change in surface area arising from these conditions was not sufficient to significantly alter the equilibrium contact angles or wetting regime. We conclude that the observed significant variation in surface wettability was more likely to depend on variation in surface chemistry than roughness; stoichiometry was the variable which most significantly influenced surface wettability, average void volume and fractional free volume, while cure temperature significantly influenced the extent of cure at both stoichiometries. Off-stoichiometry formulation and elevated ambient cure temperature significantly increased system average void volume while fractional free volume decreased, which may be significant for the barrier properties of the final coating.

## Abstract 2

The hydrophilicity of epoxy resin coatings is most sensitive to humidity and induction time. Stoichiometry (amine content) has some effect on surface and strongly influences bulk *T*_g_/void volume along with cure temperature.

## Graphical abstract

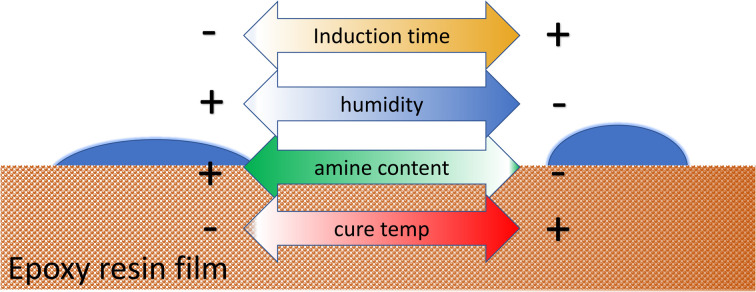

## 1. Introduction

Epoxy resins are widely used in cargo tanks on ships as a protective coating to enable the transport of reactive and corrosive cargoes. Within the coatings, composites and adhesives sectors, epoxy resin technology has long been exploited due to its favourable combination of properties including good chemical resistance, excellent mechanical properties, and low cost. Consequently, by 2024, it is projected that the global epoxy coating market will exceed $42.3 billion based on a 7.8% compound annual growth rate.^1^ With the continued increase in world maritime trade over the last decade, along with the United Nations Conference on Trade and Development (UNCTAD) projecting an increased annual average growth rate of 2.4% over the period 2022 to 2026, the demand for larger chemical tankers has increased, highlighting the importance of using suitable coatings to protect these assets.^2,3^

Liquid bulk cargoes, such as oil, oil products and various chemicals, are transported in specialised, protectively coated tankers. A single coat (typically 160 microns) could leave defects such as pinholes or pores which reach down to the steel substrate, leading to corrosion.^4^ Because pinholes only constitute a small fraction of the total area, overcoating with a second layer should reduce the risk of uncoated steel substrate. However, two coat systems may experience intercoat adhesion failure manifesting as blistering or delamination. Blisters can retain cargo, leading to leaching into subsequent loads, while delamination can result in coating degradation and cargo contamination, incurring large financial implications. The cost for full tank coating refurbishment on chemical tankers can often reach in excess of $3 million per vessel.^5^

While thermoplastic interface chemistries have been extensively studied^6–8^ and previous work has investigated epoxy–substrate interfaces,^9–12^ epoxy–epoxy interfacial chemistry remains less documented and consequently the causes of these intercoat adhesion failures in two coat systems are not fully understood. Epoxy–epoxy interface adhesion is hypothesised to be influenced by first coat surface properties. During practical application, cure conditions (induction time, ambient temperature and ambient relative humidity, RH) are often variable, and the impact on surface properties not fully understood or characterised. For strong adhesion between layers of epoxy, it is thought that some interdiffusion between the first and second layers must occur, so that the cross-linked structure propagates across the interface. Some authors suggest that roughness and surface energy are important for adhesion^13–15^ while others regard these as quite weak effects and instead propose that surface chemistry and the ability to form an interpenetrating network are more relevant.^16,17^

**Fig. 1:**
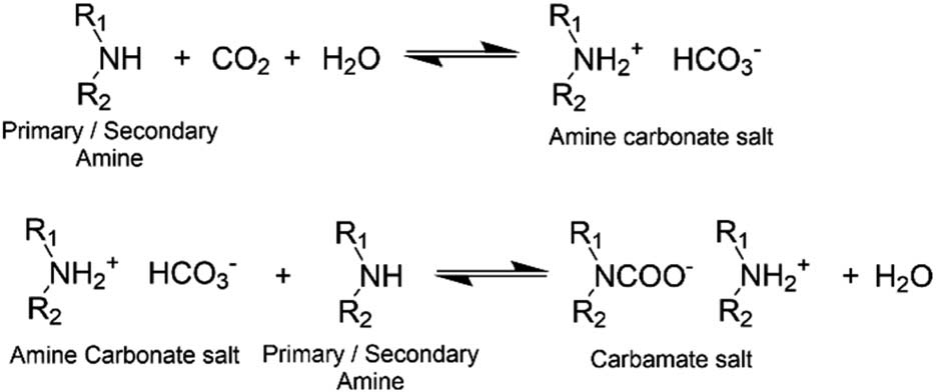
Reaction scheme of primary/secondary amine and CO_2_.^43^

Induction time: the amount of time between reactant mixing and coating application, influences mixture compatibility and viscosity. The amine component of epoxy systems is often less compatible and so tends to migrate to the film surface during the liquid cure phase.^18^ As amines are characteristically hygroscopic and efficient carbon dioxide scavengers, at the surface they can react with carbon dioxide and moisture in the air (Fig. 1). This leads to a disproportionate fraction of amine groups consumed near the sample surface and sometimes manifests as a white surface layer (carbamate) to which subsequent coats cannot properly adhere. This leads to the formation of a weak boundary layer between coats.^4,19–22^ The inclusion of an induction time prior to coating application allows free, low molecular weight amine hardener to pre-react with epoxies to produce oligomeric molecules, which improves amine-epoxy compatibility and increases bulk viscosity. This slows down any migration of amine and consequently reduces the opportunity for carbamate to form. While this is understood, for a given induction time, there are limited data quantifying the amount of surface layer formed and the subsequent impact on film surface properties.

Seasonal and geographical variation means ambient cure RH and temperature are variable. In industrial environments, RH should be maintained within the limits specified by the coating manufacturers (often <50% RH for tank linings) and ambient temperature usually falls between 25 °C–35 °C. However, it is not known how variation within these ranges influences first coat surface properties and in turn intercoat adhesion. In this article, we explore the impact of stoichiometry, cure conditions and the interplay of cure conditions (induction time, RH, temperature) on epoxy systems by systematically changing these variables. We deliberately chose two very different stoichiometries; one an idealised system in which the fraction of available amine groups is 100% of the number of epoxy groups; and can cure to completion by addition. The second system uses the same components, but in a ratio that is more typical in industry, where that amine concentration is only 35% of the epoxy concentration, and accelerators are used to promote epoxy homopolymerisation. By doing this, the effects of these variables on surface or bulk properties can be determined and the potential implications on epoxy–epoxy intercoat adhesion identified.

## 2. Experimental

### 2.1 Materials and sample preparation

**Fig. 2:**
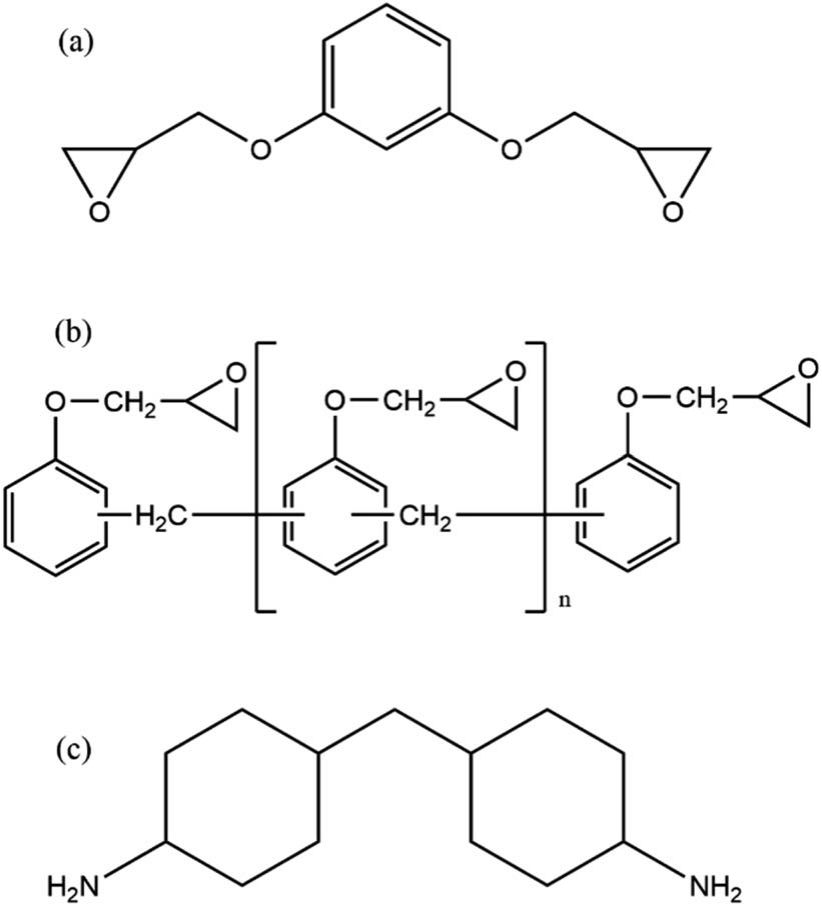
(a) RDGE (b) D.E.N. 431, *n* = 0.7 (c) PAC-M.

Dow Epoxy Novolac, D.E.N. 431 (OLIN, Missouri United States), resorcinol diglycidyl ether, RDGE (Huntsman Corporation, Texas United States) and 4,4-diaminodicyclohexylmethane, PAC-M (Evonik Industries, Essen Germany) were obtained and used as received. The nominal structures of these molecules are shown in Fig. 2. The epoxide equivalent weight (EEW) of each epoxide containing material (D.E.N. 431 and RDGE) was determined titrimetrically using ASTM-1652 (ref. 23) and molecular structure confirmed using NMR. 2,4,6-tris(dimethylaminomethyl)phenol (DMP-30), 1-methylimidazole (1 Ml) and 2-ethyl-4-methylimidazole (2-E-4-Ml) were obtained from Merck and used as received.

For all systems studied, RDGE and D.E.N. 431 were used in a 3 : 1 mass ratio, comprising the mixture's epoxide component. This epoxide component was then mixed with PAC-M at two distinct ratios to produce two stoichiometries: 100% (1 : 1 of epoxide groups to amine active hydrogens) and 35% (1 : 0.35 ratio of epoxide groups to amine active hydrogens). Each mix totalled 50 grams. In addition, 0.96 g of DMP-30, 1.37 g of 1-Ml and 0.68 g of 2-E-4-Ml was added to each 35% stoichiometry 50 g epoxy/amine mix to promote homopolymerisation of the epoxide components. These stoichiometries allow for the comparison of systems utilising different reaction mechanisms, namely step-growth amine – epoxy addition (100%) and anionic chain-growth polymerisation of epoxide groups (35%). A control third system at 100% stoichiometry with accelerators (100% + Acc.) was to enable the contribution of the accelerators to be resolved from the effects of stoichiometry. Resins and additives in the experiments and the relative ratios were all sourced from Patent submissions and MSDS's.^24,25^

After mixing, films (75 mm length × 12 mm width × 150 μm thickness) were cast onto glass slides using a cube applicator (TQC Sheen, Netherlands) and allowed to cure for 24 hours under controlled conditions.

Temperature and RH were controlled using a vacuum oven and saturated salt solutions to produce films under the condition shown in Table 1.

**Table 1:** Cure conditions of each sample

Induction time/min	Temperature/°C	RH/%
<0.5	25	40
15	25	40
<0.5	25	80
15	25	80
15	25	<5
15	35	<5
15	35	40
15	35	80

When investigating variation in ambient cure temperature (25 or 35 °C), RH was maintained at 40%. When investigating variation in RH (<5–80%) temperature was maintained at 25 °C.

### 2.2 Gravimetric analysis

Sample and substrate (glass slide) mass was recorded using a Sartorius CPA124S microbalance, precision ± 0.00005 g. The film surface (9 cm^2^) was then cleaned using a cotton bud soaked in D_2_0 to remove any carbamate (water soluble).^26^ This solution was retained for NMR analysis. Sample and substrate mass was then re-recorded, and the difference determined.

### 2.3 Atomic force microscopic analysis

AFM images were recorded using a Bruker MM8 AFM. The films were studied using PeakForce QNM mode capturing 10 × 10 μm images with 512 samples per line. NuNano Scout 350 probes with an 18 N m^−1^ spring constant and 350 kHz resonant frequency were used. Deflection sensitivity, spring constant and tip radius were determined prior to use *via* tip calibration protocol (ramp, thermal tune) using silicone and sapphire calibration standards. Images were processed and analysed using NanoScope Analysis. AFM provides two measures of surface roughness, root-mean-square surface roughness, *R*_q_, and rugosity. *R*_q_ is defined as^27^1
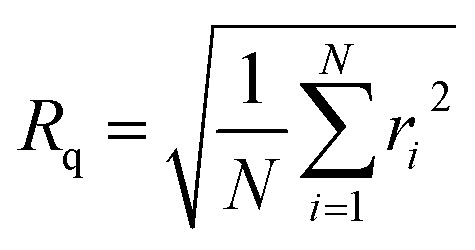
where *N* is the number of data points and *r*_*i*_ is the deviation in height of a point from the mean. Rugosity is a measure of the extent to which surface height variations increase the sample surface area:2
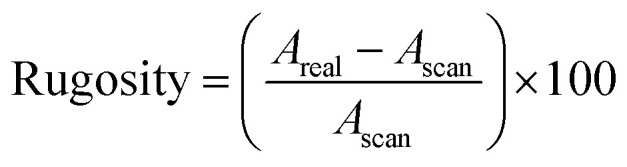
where *A*_scan_ is the area defined by the scan size and *A*_real_ is the total surface area of the sample that is scanned.

### 2.4 Contact angle analysis

Contact angle measurements were collected using the sessile drop technique and recorded using a UI-3370CP-M-GL Rev.2 camera equipped with a telecentric lens to remove the effect of field depth. 10 μL of probe liquid (UHP water, glycerol, formamide, propylene glycol, ethylene glycol or diiodomethane) was placed on the film surface and the static contact angle recorded and measured using the DropSnake plug-in on ImageJ (Fuji).

### 2.5 Dynamic mechanical analysis

A Q800 DMA was used to perform dynamic mechanical tests on samples with dimensions 40 mm × 13 mm x 3 mm (length × width × thickness). A single cantilever in DMA multi-frequency strain mode, with an amplitude of 15 μm and a frequency of 1 Hz was applied. Samples were scanned using a heat ramp at 10 °C per minute from 30 °C to 160 °C.

### 2.6 Positron annihilation lifetime spectroscopy

Positron annihilation lifetime spectroscopy (PALS) measurements were carried out at the University of Sheffield using a fast–fast coincidence circuit (50 ns).^28^ Two identical sample films (2 mm in thickness) sandwiched a ^22^Na positron source and were placed between a pair of fast plastic scintillators and photomultiplier tubes (gamma detectors) to acquire lifetime spectra. Each spectrum was collected to a minimum of 1 million counts from annihilation events and the time resolution was monitored to 470 ps.

The positron decay spectra are made up of a series of lifetimes which were resolved into three finite lifetime components: *T*_1_ (shortest lived, p-Ps 0.125 ns), *T*_2_ (free positron lifetime 0.3–0.5 ns), *T*_3_ (longest lived, o-Ps > 0.5 ns). Using the Tao-Eldrup model, which assumes voids are infinitely deep spherically symmetric potential wells, *T*_3_ can be correlated to the mean void size by first determining the medium free volume cavity radius using the empirical eqn (3):3
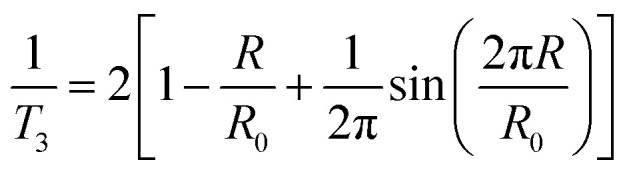
where *R* is void radius and *R*_0_ = *R* + Δ*R* where Δ*R* is 1.656 Å.^29,30^ Free volume cavity radius can then be used to calculate average void volume (eqn (4)) and fractional free volume (eqn (5)).4
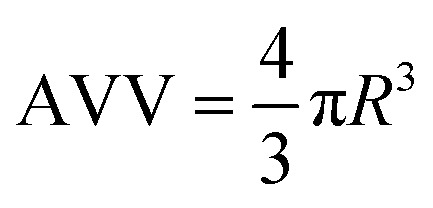

5 FFV = *I*_3_ × AVV

where AVV is average void volume, FFV is fractional free volume and *I*_3_ is the relative intensity of the o-PS annihilation lifetime (the percentage of positrons annihilating by the pickoff mechanism).^28,31^ The fitting procedures are evaluated and described in greater detail in the ESI.†

## 3. Results and discussion

### 3.1 Quantification of surface layer formation

Mass loss following wiping of cured samples provides a simple measure of the extent of surface layer formation (carbamation). Wiping samples to remove carbamate revealed no detectable change in mass (>5 × 10^−5^ g) for coatings that were allowed to cure at 25 °C, 40% RH (Table 2). However, at 80% RH there was a detectable mass loss, and the inclusion of an induction time significantly reduced this mass loss. While 80% RH exceeds what is specified in industrial environments (typically < 50%), by using a slightly larger experimental range a more detailed characterisation was obtained and potential problems resulting from loss of climate control can be identified. Our results are consistent with the works of Didas and Flaig who also reported that carbamation of amines tends to increase with increasing RH^32,33^ and highlights the impact of induction time: increasing system compatibility and mixture viscosity, leading to decreased surface layer formation Table 2.

**Table 2:** Change in mass pre and post cleaning with D_2_O of the 100% stoichiometry systems

Induction time/min	RH/%	Change in mass/mg mm^−2^
<0.5	40	0.0 ± 0.0
15	40	0.0 ± 0.0
<0.5	80	1.3 ± 0.2
15	80	0.7 ± 0.2

Attempts to characterise the chemistry of the material removed with D_2_0 using NMR were unsuccessful. This is most likely because the quantity of material extracted by this method was too small to obtain a clear spectrum and there was likely a mixture of components (carbamate, unreacted PAC-M).

### 3.2 The impact of cure chemistry and stoichiometry on surface roughness

Interdiffusion, entanglement and crosslinking are thought to be factors that influence epoxy intercoat adhesion.^34–36^ Surface wettability limits interdiffusion and entanglement as it concerns the ability of a liquid to spread and permeate cracks within a substrate. Therefore, as surface roughness influences wettability, it is important to characterise surface roughness, as a function of cure chemistry and stoichiometry, to determine the effect on wettability and wetting mechanism (Wenzel or Cassie–Baxter).

**Fig. 3:**
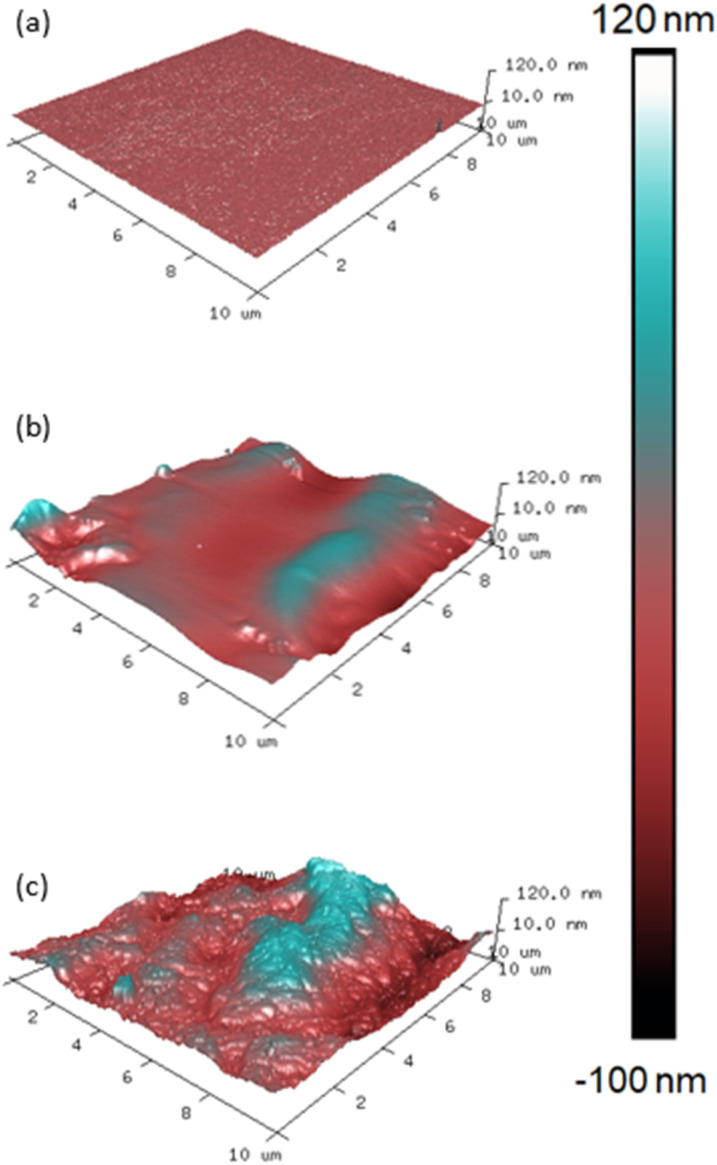
AFM Images of 100% stoichiometry films cured at (a) < 5% RH (b) 40% RH (c) 80% RH, temperature maintained at 25 °C. In each case, vertical scales have been optimised to highlight the variation in surface height.

**Fig. 4:**
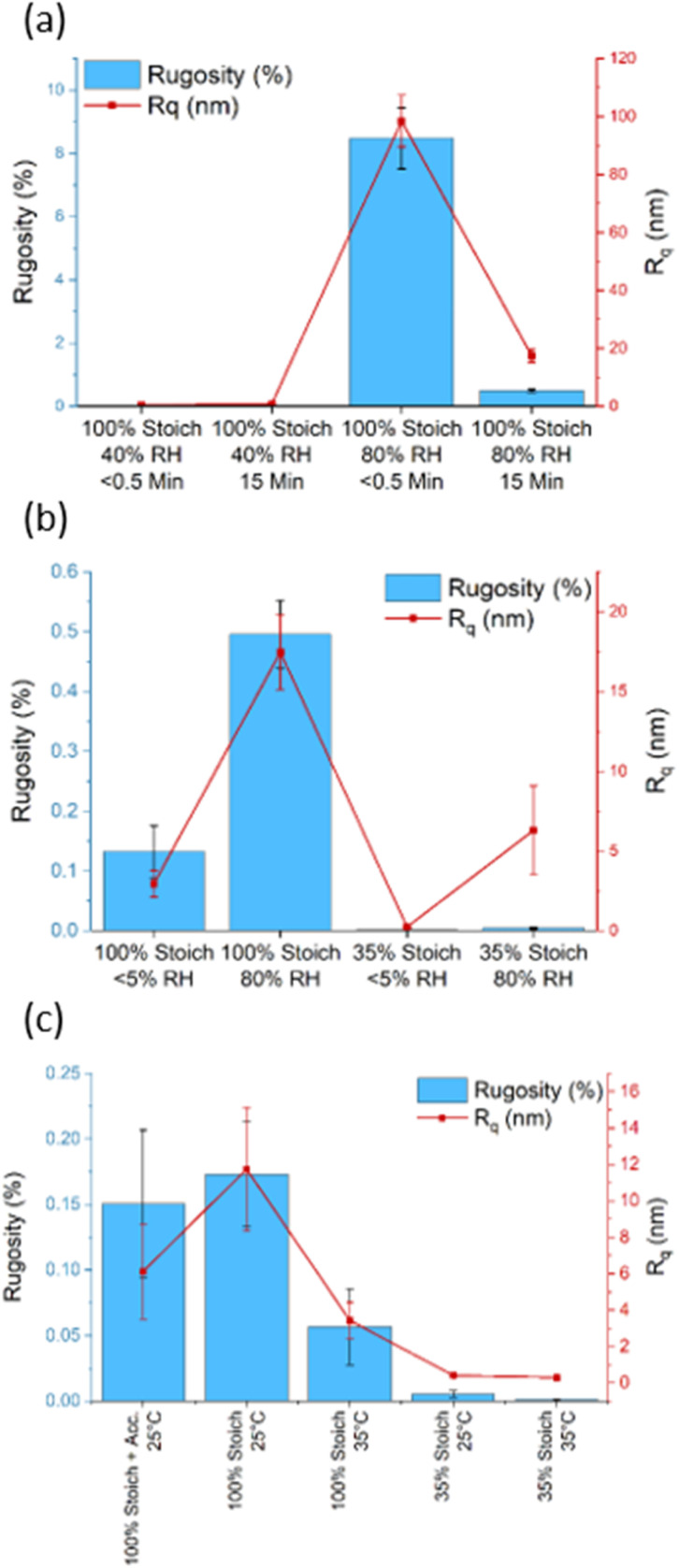
The surface roughness (R_q_/nm) and rugosity of (a) 100% stoichiometry systems, < 0.5- or 15 minutes induction time at 40 and 80% RH (b) 100% and 35% stoichiometry systems at < 5% and 80% (c) 100%, 35% and 100% with accelerators stoichiometry systems at 25 and 35 °C.

The 100% stoichiometry systems had a significantly higher *R*_q_ and rugosity than the 35% stoichiometry systems. At both stoichiometries, increasing the cure temperature from 25 °C to 35 °C decreased *R*_q_ and rugosity (Fig. 4). While significant within the precision of the AFM measurement, the rugosity never exceeded 10%. Consequently, it is unlikely to have affected surface wettability as both the 25 °C and 35 °C -cured films were already relatively smooth with *R*_q_ < 12 nm and no distinct features were observed on the μm scale (Fig. 3). No significant difference in *R*_q_ or rugosity was observed between the 100% systems with and without accelerators.

Increasing RH and decreasing induction time significantly increased surface roughness and maximum peak height (Fig. 3). This is interesting as one may expect the mixture with the lower viscosity (due to a shorter induction time) to form a flatter surface under gravity. This increase was likely due to carbamate formation which forms a weak boundary in two-layer epoxy systems and reduces intercoat adhesion.^19^ The effect of induction time remained far more apparent at 80% RH compared to 40%, Figure (a), consistent with the gravimetric analysis. In addition, the impact of increasing RH was more apparent in the 100% stoichiometry formulation than the formulation at 35% stoichiometry, Fig. 4(b). The former system contains a greater proportion of primary amine groups that are susceptible to carbamation. The quantity of carbamate formed on this scale was too low to be detected through IR analysis or NMR. Fig. 4(c) shows that for the additional control sample of 100% stoichiometry with added accelerators, the surface roughness measures are not significantly altered by the accelerators.

### 3.3 The impact of cure conditions and stoichiometry on surface free energy (SFE) and wettability

**Fig. 5:**
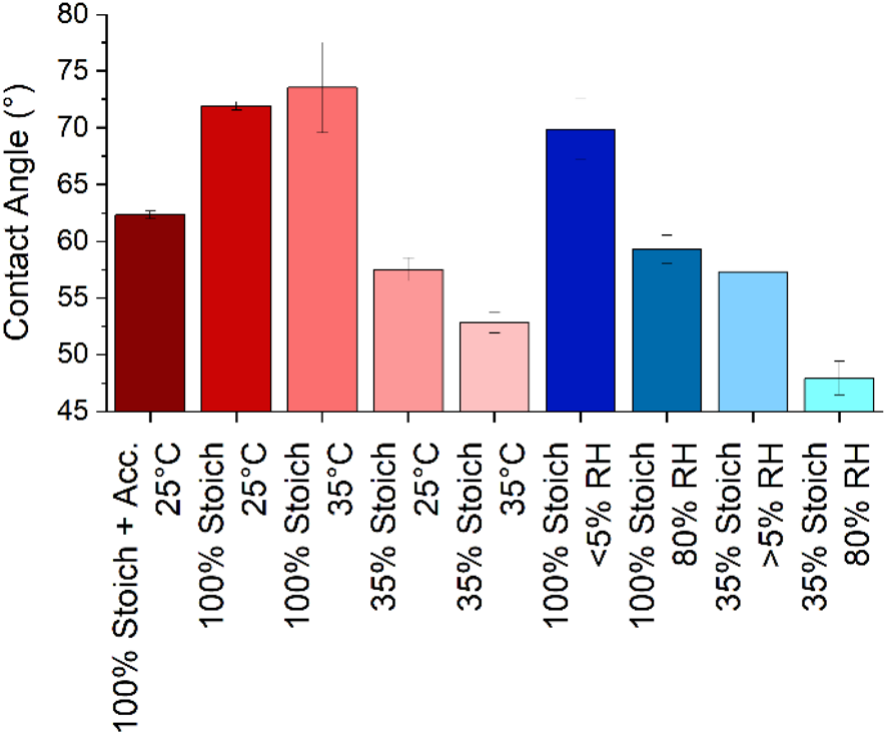
The static water contact angle (at ∼10 seconds) of each epoxy system and uncertainty. Cure processes were 25 °C and/or 40% RH unless otherwise stated.

Determining wettability and SFE as a function of cure chemistry and stoichiometry will give insight into the likely ability of a second coat of epoxy, or other material to spread upon the first coat during application and permeate any cracks that may be present in the substrate. This is of interest as increased surface wetting increases the surface area for interdiffusion and interface formation. Three measures of wettability or surface free energy (SFE) characterisation were considered; water contact angle (WCA), Zisman plots and the Owens–Wendt method. While WCA cannot determine surface energy, it does offer a convenient measure of surface wettability. Only the WCA of the 35% stoichiometry films were significantly affected with an increase in cure temperature, increasing hydrophilicity (Fig. 5). However, when samples of either stoichiometry were cured under high RH, at both stoichiometries, the resulting film surfaces were more hydrophilic than those cured at lower RH. While greater surface wettability is usually desirable in overcoated systems, this is likely due to the presence of water-soluble carbamate on the surface as humidity promotes carbamation.^32,33^

The most significant difference in surface wettability was that between the two stoichiometries with the 35% systems being more hydrophilic than the 100% systems. System rugosity, measured using AFM, was used to estimate the impact of roughness on equilibrium contact angles using the Wenzel correction:

6 cos *θ*_rough_ = *r* cos *θ*_smooth_

where *r* = (1 + rugosity/100).^37^ For these films where contact angles are < 90°, contact angles decrease with increasing *r*. Application of the Wenzel correction showed that the change in WCA was typically < 0.1°, indicating that the difference in surface wettability was due to chemical rather than physical differences.

**Fig. 6:**
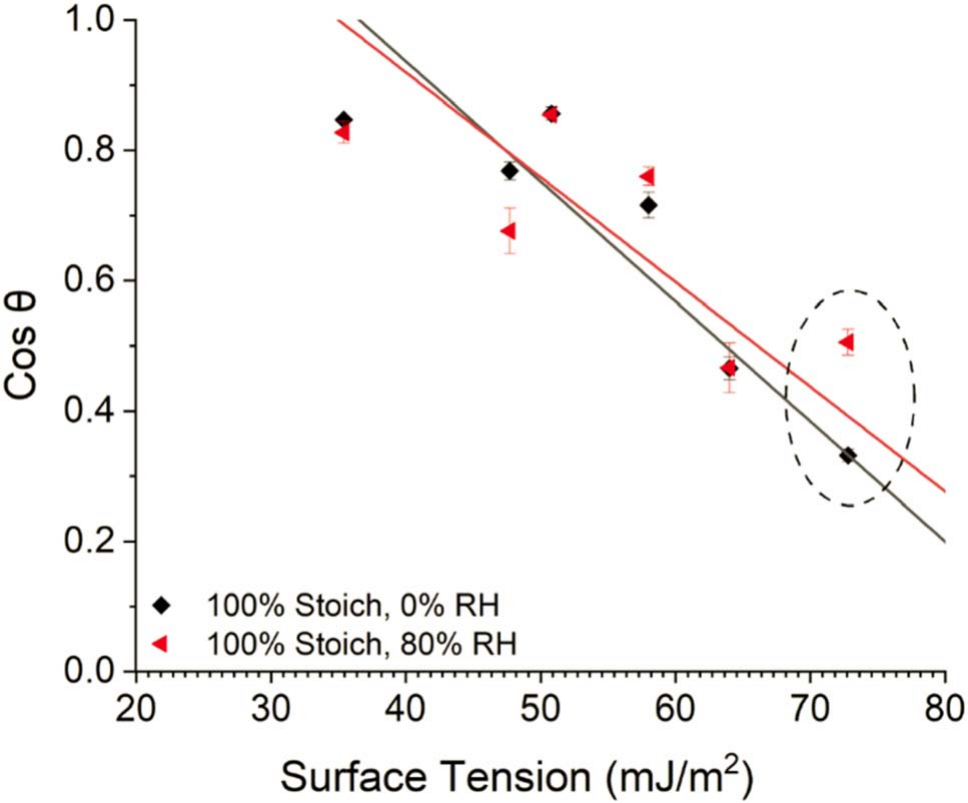
Zisman plot of the 100% stoichiometry system cured at <5% or 80% RH, 25 °C. The points associated with WCA have been circled.

Zisman plots (Fig. 6) were produced to estimate the SFE of each system and to determine if the observed large variation in WCA between high and low RH cured systems was due to differences in SFE or as a result of water changing the surface. The plots were produced by plotting the measured cosine contact angle of a series of solvents, against respective literature obtained surface tension values, and extrapolating a least-squares regression line to the point where cos *θ* = 1, as here surface tension is equal to SFE.

Increased cure RH caused a much greater decrease in contact angle (increase in cos *θ*) of water compared to all other solvents analysed (Fig. 6, circled). This showed that water caused a surface change and ultimately led to large, propagated error upon SFE calculations, limiting interpretation. Therefore, the Owens–Wendt model was instead utilised to determine the SFE of each system. The three solvents used for Owens–Wendt model analysis were selected due to their varying polar : dispersive surface energy contributions (glycerol – 30 : 34 mJ m^−2^, formamide – 27 : 31.4 mJ m^−2^, diiodomethane – 0 : 50.8 mJ m^−2^) providing for more complete analysis.

**Fig. 7:**
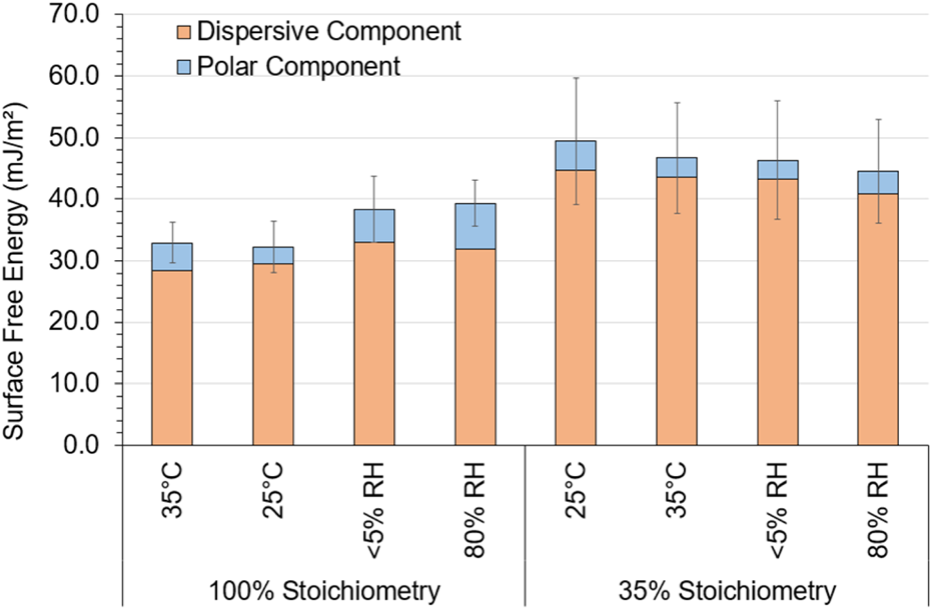
Owens–Wendt model determined dispersive and polar surface energy of each epoxy system and uncertainty; probe liquids used were glycerol, formamide, diiodomethane. Cure conditions were 25 °C and 40% RH unless otherwise stated.

Fig. 7 displays the SFE of each system derived using the Owens–Wendt model. The Owens–Wendt model provides an estimation of the total surface energy though calculating the polar and dispersive surface energy components. As shown in Fig. 7, variation in the polar, dispersive, or total SFE, attributed to varying ambient cure chemistry and conditions, was low compared to the relatively large, propagated error indicating no significant impact of varying ambient cure conditions on SFE.

### 3.4 The impact of cure conditions and stoichiometry on system *T*_g_ and modulus

The extent of cure (or conversion achieved) of an epoxy system, rarely complete in practical coating applications, is thought to be important for barrier properties, flexibility, and surface properties. Within epoxy resin chemistry, system *T*_g_ can give an indication of the conversion achieved, in same stoichiometry systems where the number of competing reactions is consistent.^38^ Once significant conversion has been achieved, a crosslinked network may be formed, depending on the system. Given epoxy–epoxy interfaces are thought to form through interdiffusion and a percolating network of bonds,^34–36^ elevated crosslink density in the first coat would be expected to decrease second coat diffusion potential thus decreasing interfacial width forming a smaller boundary layer. Therefore, it is important to characterise ambient-cure system *T*_g_ as a function of cure chemistry prior to interface investigation.

**Fig. 8:**
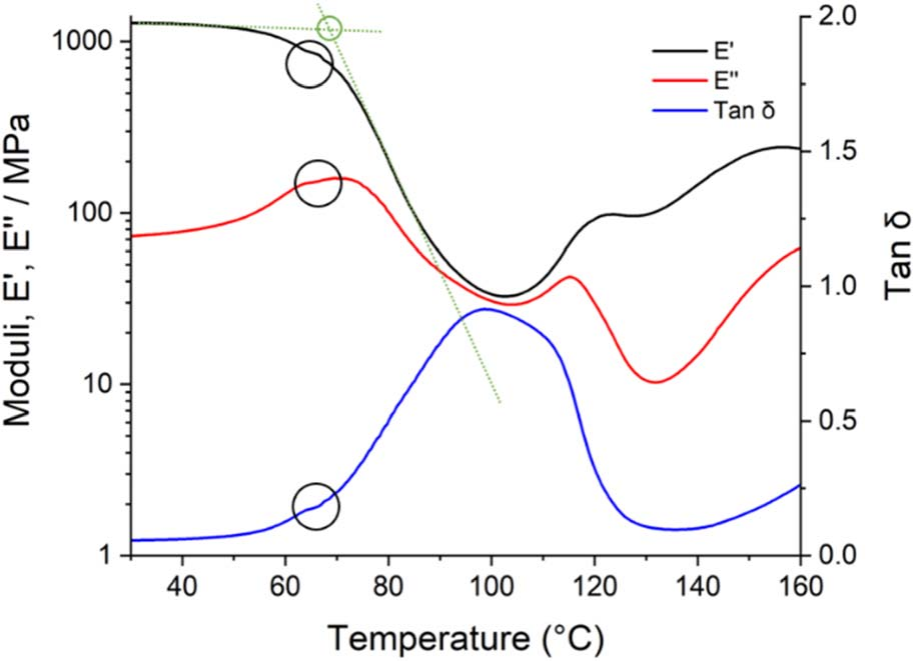
Storage modulus *E*′, loss modulus *E*′′, and tan *δ* against temperature of a 35% stoichiometry system detailing onset ambient cured *T*_g_ (green intercept) and evidence of *in situ* curing (black circles).

The decay in the storage modulus, calculated by the intersection of the two linear regions before and after the drop in storage modulus (Fig. 8), was used as a comparative measure of conversion achieved as a function of cure condition. This is not a direct measure of ambient cured system *T*_g_ as the heating involved in the DMA experiment will inevitably increase epoxy conversion during the experiment as evidenced by the slight levelling off seen in the *E*′, *E*′′ and tan *δ* at approximately 65 °C in Fig. 8. For an inert system, a heating rate of 3 °C per minute would typically be used to ensure thermal equilibrium. Here, however, a higher heating rate of 10 °C per minute was used to minimise the extent of curing during the measurement (Table 3). This procedure incurred a systematic error due to the temperature lag between apparatus and sample. Although it is clear that *T*_g_ changes over the time scale of DMA experiments and results are offset by changing heating rate, both of these effects are systematic in nature, and our separate experiments using a polystyrene standard (ESI†) show that consistent *T*_g_ measurements are possible if heating rate is consistent (±0.8 °C).

**Table 3:** DMA analysis of each epoxy system detailing onset ambient *T*_g_ and storage modulus

Stoichiometry (%)	Cure temperature (°C)	Cure RH (%)	Decay in storage modulus (°C)	Storage modulus
100	25	40	67.6 ± 1.9	1150 ± 120
100	35	40	78.3 ± 2.1	1070 ± 210
100	25	< 5	66.1 ± 3.5	1150 ± 200
100	25	80	66.8 ± 5.7	1520 ± 720
35	25	40	80.5 ± 2.0	930 ± 210
35	35	40	95.4 ± 2.7	730 ± 310
35	25	< 5	72.3 ± 4.8	960 ± 290
35	25	80	84.1 ± 3.6	920 ± 340

Accurate comparisons of *T*_g_ cannot be made between the two different stoichiometries as the systems have different competing curing reactions. Unlike the 100% stoichiometry systems, the 35% stoichiometry systems are in epoxide excess and contain accelerators which promote anionic chain-growth polymerisation of epoxide groups (epoxy homopolymerisation). While epoxy homopolymerisation can increase crosslink density, and therefore *T*_g_, through intramolecular etherification,^39–41^ the activation energies of the competing reactions may not be the same, and thus a higher *T*_g_ in an epoxide excess system with two competing reactions need not imply a higher conversion of epoxy groups. At both stoichiometries, variation in ambient temperature significantly increased the temperature of decay in the storage modulus (Table 3). This suggests that systems cured at higher ambient temperatures may have an elevated crosslink density and so be less favourable for second coat interdiffusion forming a smaller boundary layer.

### 3.5 The impact of cure conditions and stoichiometry on system average void volume and fractional free volume

Positron annihilation lifetime spectroscopy (PALS) allows for accurate estimations of polymer average void volume, AVV (mean free volume void size) and fractional free volume, FFV (total amount of free volume within a sample/free volume fraction) by measuring electron induced positron annihilation lifetime inside a free volume void and using this measurement to determine free volume void size (eqn (3) and (4)).^29,30^ Previous studies have shown a correlation between increased AVV/FFV and increased rate/extent of solvent ingress.^28^ However, far less has been documented regarding the impact of AVV and FFV on the potential for second coat interdiffusion. In particular, penetrant molecular volume associated with second coat interdiffusion (D.E.N. 431: ∼584 Å^3^/RDGE: ∼305 Å^3^/PAC-M: ∼368 Å^3^) would be much larger than previously characterised probe solvents (methanol: ∼67 Å^3^) and the rate of ingress has been shown to significantly decrease when penetrant volume exceeds AVV.^28,42^

**Fig. 9:**
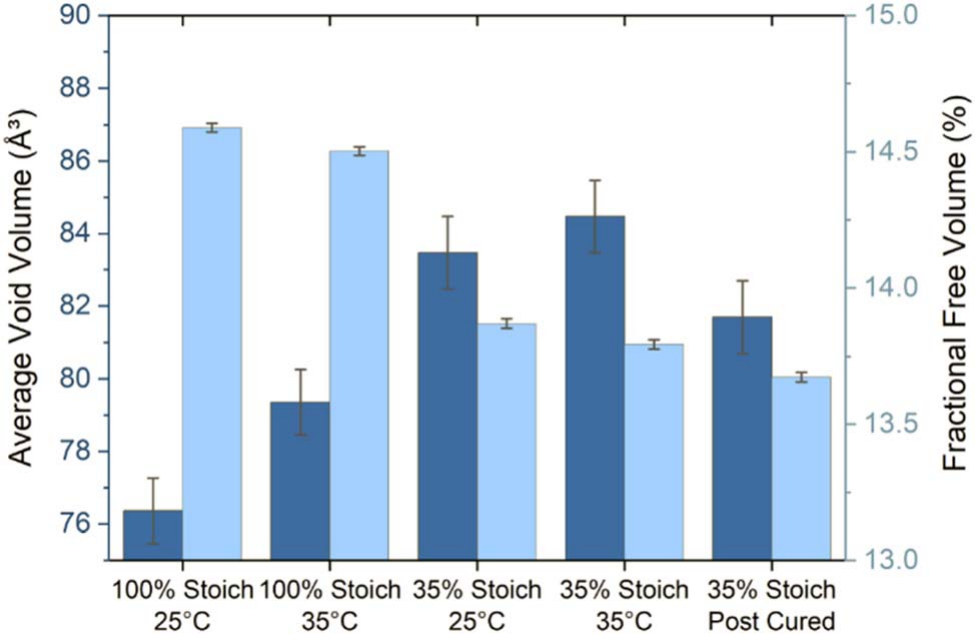
Average void volume (dark) and fractional free volume (light) of each epoxy system.

Increasing the cure temperature from 25 °C to 35 °C significantly decreased FFV in the 35% system. However, it both increased AVV and decreased FFV in the 100% system (Fig. 9). A similar pattern was seen as a function of stoichiometry, as the 35% system recorded a higher AVV, but lower FFV compared to the 100% system. Since the AVV and FFV are not proportional to one another, this indicates that the number of voids detected by PALS is very sensitive to the cure conditions applied. This result could be interpreted as the increase in cure temperature or use of a 35% stoichiometry led to a smaller number of larger voids (increasing AVV), while the overall fraction of free volume was lower (decreasing FFV). Currently it is not fully understood which property, AVV or FFV, is more influential in promoting intercoat diffusion; previous studies have detailed the importance of both properties in extent of penetrant ingress.^28,42^

AVV ranges from ∼76–85 Å^3^, which is significantly smaller than the calculated molecular volume of the second coat penetrants. Therefore, it is expected that the rate of and ultimate extent of interdiffusion upon overcoating will be relatively slow forming a narrow interface. The results may imply that the first coat may require some kind of surface plasticisation (*e.g.*, by the second coat) to increase AVV in order to allow second coat penetration, or perhaps that the bulk AVV, which is measured using PALS, is not consistent with surface AVV.

## 4. Conclusions

Factors affecting amine cured resin surfaces such as carbamation are known to influence their performance as substrates for adhesion, but can be challenging to characterise with bulk chemistry techniques such as NMR, or with IR. We have found that for a variety of controlled conditions, and their combinations, it is possible to detect early stages of carbamation using AFM and contact angle analysis. The most important single parameter appears to be RH, and its impact is most noticeable in formulations with high amine content.

WCA results indicated that the low amine content resin systems were most hydrophilic. Since these resins also have the higher *T*_g_ values, and low roughness, it appears unlikely that this difference can be attributed to either reorientation of surface molecules or to surface topography; therefore, surface chemistry is most likely responsible for the greater wettability.

Increasing the cure RH significantly increased surface roughness, surface layer formation, and wettability, but increasing the cure temperature had less of an effect on the surface properties. Noticeably, although hydrophobicity could be sensitive to curing conditions, the apparent surface free energy is little affected. This indicates that WCA alone is a flawed measure of surface properties in these systems, but may provide a convenient indication of carbamation.

Cure temperature more significantly affected the bulk properties compared to RH, with increasing cure temperature increasing extent of cure, FFV while decreasing AVV. This suggests film bulk properties are influenced more by cure temperature whereas surface properties are more significantly impacted by RH.

The material properties (*R*_q_, rugosity, wettability, *T*_g_, AVV, FFV) characterised in this study are hypothesised to be influential in intercoat adhesion and show statistically significant variation when ambient cure conditions and stoichiometry are varied. This indicates that in order to optimise interdiffusion and achieve strong second coat binding, the cure conditions and chemistry utilised should be considered, but physical topography has limited influence.

## Conflicts of interest

There are no conflicts to declare.

## Supplementary Material

RA-012-D2RA05067F-s001
