# Supplementary material for: The influence of ambient cure chemistry and stoichiometry on epoxy coating surfaces
Source: RSC Adv. 2022 Oct 10;12(44):28746–54. doi: 10.1039/d2ra05067f (PMC9549571; doi:10.1039/d2ra05067f)
Supplement: RA-012-D2RA05067F-s001 [file RA-012-D2RA05067F-s001.pdf]

Electronic Supplementary Material (ESI) for RSC Advances.
This journal is © The Royal Society of Chemistry 2022

Supporting Information

The Influence of Ambient Cure Chemistry and Stoichiometry on Epoxy Coating Surfaces

Callum Bannister,*[a] Alan Guy,[b] Ralitsa Mihaylova,[b] Joseph Orgill,[c] Stephanie Burg[c] ,Andrew Parnell,[c] Stephanie L Burg,[c] and Richard L Thompson,*[a]

Materials Chemistry Department, Durham University, Durham, UK.

Corresponding Author:

E-mail: r.l.thompson@durham.ac.uk

E-mail: callum.c.bannister@durham.ac.uk

Table 1: T_g of a polystyrene standard (M_W = 95,000) determined using DMA at different heating rates.

Heating Rate (°C)	T_g (°C)			$\bar{x}$	σ
	1	2	3		
3	126.2	126.2	125.6	126.0	0.3
10	141.8	143.0	143.2	143.7	0.8

As there is no standardized approach for fitting PALS data [1] a custom python script was used to fit the data and to calculate the lifetimes (script available upon request). Using different fitting software / minimization algorithms can change the absolute values of the lifetime results and consequently the free volume values, but the trends between the samples remain consistent. Two least squares fit modes were compared (Trust Region Reflective & Levenberg-Marquardt), along with a stand-alone PALS fitting software (DQuickLTFit v4.1)[2]. Results from the 3 different fitting approaches are shown in the figure below. For the data presented in the main paper the Trust Region Reflective algorithm was used.

As can be seen the shift in magnitude is small and they are all similar, n.b. the y axis scale.

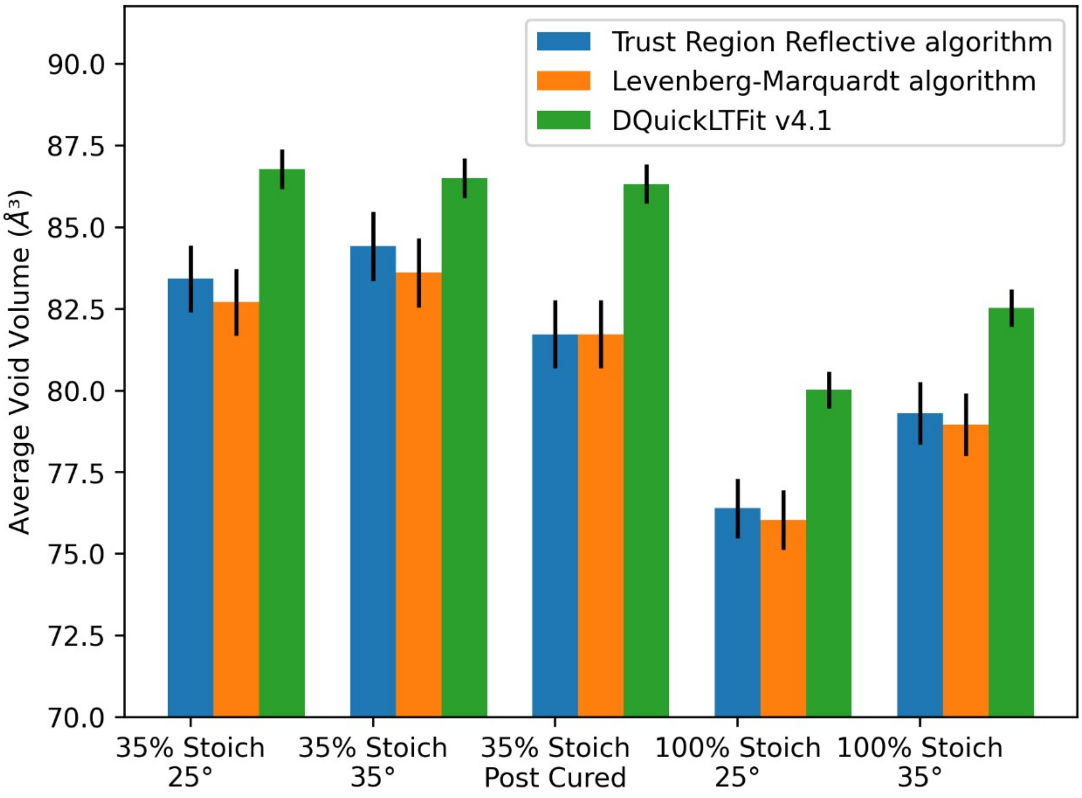

Figure 1. A comparison of three different approaches to fitting the decay lifetimes of the ortho-positronium signal, the signal responsible for probing the free volume in the different epoxy samples.

<u>References</u>

1.) D. Petschke, R. Helm, T. E. M. Staab, Data in Brief, 2019, **22**, 16-29

2.) D. Petschke, dpscience/DQuickLTFit: DQuickLTFit v4.1, 2019, DOI: 10.5281/zenodo.3356830
